# Surrogate broodstock to enhance biotechnology research and applications in aquaculture

**DOI:** 10.1016/j.biotechadv.2021.107756

**Published:** 2021

**Authors:** Ye Hwa Jin, Diego Robledo, John M. Hickey, Mike J. McGrew, Ross D. Houston

**Affiliations:** The Roslin Institute, University of Edinburgh, Easter Bush Campus, Roslin EH25 9RG, UK

**Keywords:** Aquaculture, Aquatic genetic resources, Breeding, CRISPR, Fish farming, Genome editing, Genomic selection, Germ cells, Sterilisation, Surrogate broodstock

## Abstract

Aquaculture is playing an increasingly important role in meeting global demands for seafood, particularly in low and middle income countries. Genetic improvement of aquaculture species has major untapped potential to help achieve this, with selective breeding and genome editing offering exciting avenues to expedite this process. However, limitations to these breeding and editing approaches include long generation intervals of many fish species, alongside both technical and regulatory barriers to the application of genome editing in commercial production. Surrogate broodstock technology facilitates the production of donor-derived gametes in surrogate parents, and comprises transplantation of germ cells of donors into sterilised recipients. There are many successful examples of intra- and inter-species germ cell transfer and production of viable offspring in finfish, and this leads to new opportunities to address the aforementioned limitations. Firstly, surrogate broodstock technology raises the opportunity to improve genome editing via the use of cultured germ cells, to reduce mosaicism and potentially enable in vivo CRISPR screens in the progeny of surrogate parents. Secondly, the technology has pertinent applications in preservation of aquatic genetic resources, and in facilitating breeding of high-value species which are otherwise difficult to rear in captivity. Thirdly, it holds potential to drastically reduce the effective generation interval in aquaculture breeding programmes, expediting the rate of genetic gain. Finally, it provides new opportunities for dissemination of tailored, potentially genome edited, production animals of high genetic merit for farming. This review focuses on the state-of-the-art of surrogate broodstock technology, and discusses the next steps for its applications in research and production. The integration and synergy of genomics, genome editing, and reproductive technologies have exceptional potential to expedite genetic gain in aquaculture species in the coming decades.

## Highlights

• Genetic improvement in aquaculture species has a major role in global food security. Advances in biotechnology provide new opportunities to support aquaculture breeding.
• Advances in biotechnology provide new opportunities to support aquaculture breeding.
• Donor-derived gametes can be produced from surrogate broodstock of several aquaculture species.
• Surrogate broodstock technology provides new opportunities for application of genome editing.
• Surrogate broodstock can accelerate genetic gain, and improve dissemination of elite germplasm.

## 1 Introduction

Aquaculture is playing an ever-increasing role in meeting the global food and nutrition demand of a rapidly growing human population (Costello et al., 2020). While there is significant scope for expansion of production to meet this demand, several pertinent challenges remain. When compared to most crop and livestock production systems, aquaculture is at a comparatively formative stage and is a relatively high-risk industry. The sustainability of aquaculture is often hindered by difficulty in fully controlling species' reproduction cycles, and the constant threat of infectious diseases which can cause major losses of stock, in addition to environmental impacts (Gephart et al., 2020). However, the application of the latest scientific and technological advances is occurring rapidly, and continued innovation is required to address the existing production barriers and support the sustainable growth of aquaculture.

While terrestrial livestock and crops have been undergoing domestication and genetic improvement for many generations, most aquaculture species remain closely related to their wild ancestors (Teletchea and Fontaine, 2014). Domestication of aquaculture species is still ongoing, and in some cases occurs in parallel to the establishment of breeding programmes (Teletchea and Fontaine, 2014). The early-stage of the domestication process is likely to correspond to high levels of natural genetic variation in farmed populations, or base populations used to establish breeding programmes. This variation can be exploited by genetic and breeding technologies to improve traits of interest, which will have a major role in supporting the sustainable growth of aquaculture production (Houston et al., 2020). The reproductive biology of aquatic species typically involves high fecundity and external fertilisation; features which make them highly amenable to application of genetic improvement and biotechnologies, including via rapid dissemination of improved genetics for impact on production (Houston et al., 2020). Recently, well-managed selective breeding programmes have been successfully applied for many high-value species and – typically augmented by the application of genomic tools – have been highly successful in making cumulative and permanent improvement of production traits (Gjedrem and Rye, 2018). However, the gains that can be made by selective breeding are limited by the heritability of the target traits, the generation interval of the species (several years for most species; Table 1), and the need to simultaneously target multiple traits in the breeding goal. In addition, advanced breeding programmes are typically closed systems, and are limited to the standing genetic variation in the broodstock (typically sourced from a limited sample of wild populations), and new variation that arises from de novo mutations. Genome editing offers major possibilities to make step improvements in production traits, overcoming some of these limitations to selective breeding (Gratacap et al., 2019). However, barriers to realising the potential of genome editing for aquaculture research and production include technical issues (such as high levels of mosaicism in edited animals), and practical barriers to application in production. These include the challenges of integration of genome editing technologies into existing breeding programmes, and the varying regulatory and public perception landscape across the world.

**Table 1:** The average generation times of selected major aquaculture species.

Family	Species	Typical generation time (years)^a^	Notes	Reference
Cyprinidae	Common carp	1–4		Jeney and Bekh (2020)
	Grass carp	4–7		Billard (1999)
	Silver carp	4–6		FAO (2005a)
	Bighead carp	5–6		FAO (2004a)
	Tench	2–4		Billard (1999)
Cichlidae	Nile tilapia	0.5		FAO (2005b)
Ictaluridae	Channel catfish	2–3		Gjedrem (2005)
Salmonidae	Atlantic salmon	3–4		Hattori et al. (2019)
	Coho salmon	2		Gjedrem (2005)
	Chinook salmon	2–3		Gjedrem (2005)
	Masu salmon	2–3		Okutsu et al. (2007)
	Rainbow trout	1–2		Hattori et al. (2019)
Sparidae	Gilthead seabream	2–3	Sequential hermaphrodite, Protandrous	FAO (2005c)
Moronidae	European seabass	2–3		FAO (2005d)
Serranidae	Orange-spotted grouper	2–5	Sequential hermaphrodite, Protogynous	FAO (2010)
Carangidae	Yellowtail	3–5		Morita et al. (2015)
	Jack mackerel	1		Morita et al. (2015)
Scombridae	Pacific bluefin tuna	3–5		Ichida et al. (2017)
Chanidae	Milkfish	5		FAO (2007)
Mugilidae	Flathead grey mullet	2–3		FAO (2006a)
Scophthalmidae	Turbot	2–3		FAO (2005e)
Pleuronectidae	Atlantic halibut	4–6		Gjedrem (2005)
Gadidae	Atlantic cod	2–4		FAO (2004b)
Tetraodontidae	Tiger puffer	2–3		Yoshikawa et al. (2020)
	Grass puffer	0.8–2		Yoshikawa et al. (2020)
Acipenseridae	Siberian sturgeon	18–28		Pšenička et al. (2015)
	Sterlet	3–9		Froese and Pauly (2019)
Crustaceans	Whiteleg shrimp	1		FAO (2006b)
	Prawn (*Macrobrachium*)	1		Gjedrem (2005)
Molluscs	Blue mussel	1–2		FAO (2009)
	Oyster	1–4	Sequential hermaphrodite, Protandrous	Bayne (2017), Bernard (1975)
	Hard clam	2–3		FAO (2004c)
	Scallops	2–3	Simultaneous hermaphrodite	Gjedrem (2005)
	Abalone	> 3		FAO (1990)

a Note that the generation interval varies depending on environmental factors, in particular temperature. Also note that the figures shown may vary according to sex, and can be considered as an average without targeted environmental manipulation to accelerate sexual maturity.

Surrogate broodstock technology involves the production of donor-derived gametes in surrogate parents, and comprises transplanting germ cells of donor animals into sterilised recipients (the surrogates), either of the same or a related species (Yoshizaki and Yazawa, 2019). Notably this technology has been applied in several high value aquaculture species, demonstrating successful inter-species germ cell transfer, fertilisation, and production of viable offspring (Yoshizaki and Yazawa, 2019). While at an early stage in terms of practical applications, surrogate broodstock technology holds major potential as a research tool, and to expedite genetic improvement in aquaculture. Specifically, surrogate broodstock can support use of genetic technologies and resources in aquaculture by (i) enabling research applications of genome editing to overcome existing limitations, (ii) shortening the effective generation interval in aquaculture breeding programmes, (iii) facilitating dissemination of tailored and potentially edited production animals of high genetic merit for farming, (iv) retaining genetic resources of both endangered and commercially important species together with cryopreservation technology of germ cells, (v) producing gametes of species which are difficult to rear in captivity in easier-to-breed recipient species. This review focuses on the state-of-the-art of surrogate broodstock research, and how the synergy of genomics, genome editing, and surrogate broodstock technologies have the capacity to expedite genetic improvement of aquaculture species.

## 2 Surrogate broodstock technology

**Fig. 1:**
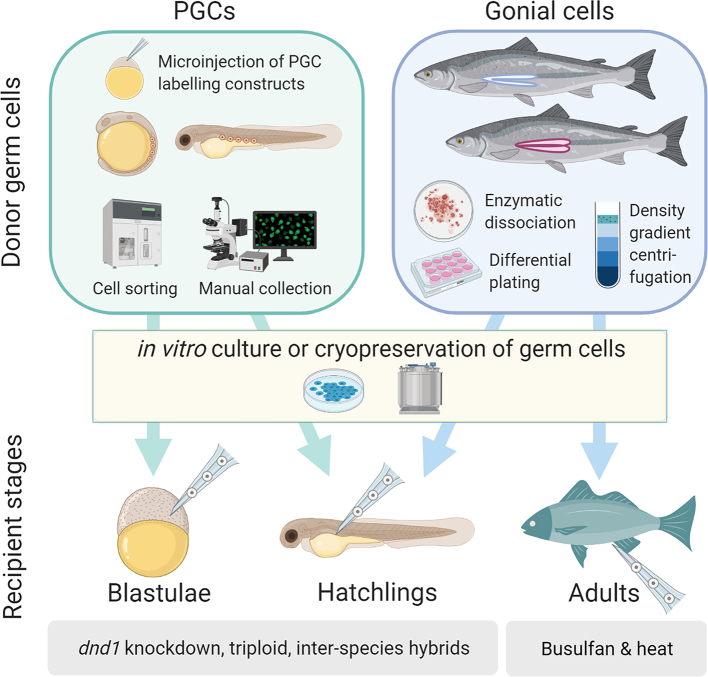
Overview of transplantation of donor germ cells (PGCs or gonial cells) at different recipient stages (blastulae, hatchling or adult stages). Labelled PGCs (by zygote microinjection of chimeric mRNA) can be isolated and enriched from somite-stage embryos or hatchlings using a cell sorter or by manual collection under microscope. Gonial cells (spermatogonia or oogonia) can be obtained either by enzymatic dissociation and filtration of gonads, or by density gradient centrifugation and differential plating to reduce gonadal somatic cells. The isolated donor germ cells can be cultured in vitro before transplantation, although not always necessary in the case of gonial cells. If needed, the isolated donor germ cells or tissues (gonadal ridges containing PGCs or whole testes / ovaries) can be cryopreserved for long term storage. PGCs can be transplanted into the marginal region of the blastodisc at the blastula stage, or into the peritoneal cavity of hatchlings in which sterility was induced, for example by knockdown of dead end 1 (dnd1), triploidy, or hybridisation. Gonial cells can be transplanted into either the peritoneal cavity of hatchlings or the gonads of adult fish through urogenital papilla. The adult stage recipients can be prepared by suppressing endogenous gametogenesis using busulfan and heat treatment.

Surrogate broodstock are sterilised recipient animals which produce gametes derived from another individual after germ cell transplantation. Surrogate broodstock technology is comprised of two main steps: a) isolation and enrichment of the precursors of gametes: germline stem cells (GSCs), and b) transplantation of GSC into sterile recipients (Fig. 1, Pšenička and Saito, 2020). The state-of-the-art GSC transplantation in aquaculture species is discussed below.

### 2.1 Isolation and enrichment of donor germ cells

There are two main types of GSCs that can be isolated and transplanted into surrogate hosts: primordial germ cells and gonial cells. The former can be isolated from embryos, whereas the latter can be isolated from sexually differentiated animals (Fig. 1). The isolation of these GSCs is the first step in applications of surrogate broodstock technologies, and methods for isolating, culturing and transplanting these cell types are outlined below.

#### 2.1.1 Primordial germ cells (PGCs)

In the main groups of aquaculture species (teleost fish, *Bivalvia* and most *Crustacea*) PGCs are specified by maternal germplasm and set aside during the cleavage stage (Extavour and Akam, 2003; Yamaha et al., 2010). The PGCs then migrate into the gonadal anlagen during embryogenesis and coalesce with gonadal somatic cells, eventually generating the gametes (Cinalli et al., 2008; Raz, 2003). To isolate and enrich PGCs from embryos, PGCs are typically labelled (either permanently or temporarily) with reporter proteins and sorted based on the reporter signals. In rainbow trout (*Oncorhynchus mykiss*), PGCs have been successfully isolated from transgenic trout carrying green fluorescent protein (*gfp*) driven by the *vasa* gene promoter (*vasa* is expressed exclusively in germ cells in animals) (Kobayashi et al., 2007; Takeuchi et al., 2004) (Table 2). This reporter labelling is promising for mass isolation of PGCs and has enabled extensive study of GSC transplantation, especially in salmonid fish. However, for some species where generation of transgenic lines is challenging, there are alternative methods for PGC labelling. The most widely used approach is the injection of chimeric mRNA consisting of a reporter protein (e.g. GFP) combined with the 3’ UTR of germline specific genes such as *vasa* or *nanos3* into the zygote cytoplasm. This use of the germline specific regulatory sequence ensures that the reporter gene is only expressed in PGCs (the construct is degraded in somatic cells; Yoshizaki et al., 2005; Saito et al., 2008). In addition, in Actinopterygii species, such as sturgeon, PGCs are formed in the vegetal pole, and therefore injection of a cell tracer dye such as FITC-dextran into the vegetal pole can successfully label PGCs (Saito and Psenicka, 2015). For species that cannot be (easily) bred in captivity and/or their embryos cannot be caught (such as deep sea water fish), it is implausible to label PGCs at the early cleavage stage, nor to produce transgenic animals, and therefore the focus for these species should be on gonial cells (see next section).

**Table 2:** Summary of germ cell transplantation in fish with different life stages of recipient, type of donor germ cells and sterilisation methods.

Recipient stage	Donor cell type	Isolation method	Donor-derived sperm (time^a^)	Donor-derived eggs (time^a^)	Sterilisation method of recipient	Transplantation method	Frequency of germline chimera formation (%)^b^	Germline transmission rate (%)^c^	Allogenic/ xenogenic (donor, D; recipient, R)	Reference
Blastula	PGC	Lower part of donor blastoderm was dissected	Yes	No	Hybrid	Lower part of donor blastoderm was transplanted to the central part of the host blastoderm	Male, 100 (15/15)	All sperm were exclusively derived from donor but no progeny test was done	Xenogenic (D, goldfish; R, goldfish x common carp hybrid)	Yamaha et al. (2003)
Blastula	PGC	Microinjection of GFP-*nanos3* 3’UTR mRNA and manually collection of GFP positive PGCs	Yes	Yes for intra-genus (pearl danio and zebrafish)	*dnd1* KD	Microinjection into marginal region of blastodisc	Male, 94 (15/16); female, 66 (2/3)	Sperm, 100; eggs, 100	Xenogenic (D, pearl danio, goldfish or loach; R, zebrafish)	Saito et al. (2008)
Hatchling	PGC	GFP positive PGCs from tg(p*vasa-*GFP)	Yes (1 yr)	Oogenesis (ovulation not reported)	No	Microinjection into peritoneal cavity of hatchling	Male, 13.5 (5/37)	Sperm, 0.4	Xenogenic (D,rainbow trout; R, masu salmon)	Takeuchi et al. (2004)
Hatchling	PGC	PGCs from cryopreserved genital ridge of tg(p*vasa-*GFP)	Yes (2–3 yr)	Yes (2–3 yr)	No	Microinjection into peritoneal cavity of hatchling	Male, 7.8 (5/64); female, 9.1 (4/44) (cryopreserved PGCs for 1 day)	Sperm, 2–13.5; eggs, 0.1–3.3	Allogenic (rainbow trout)	Kobayashi et al. (2007)
Hatchling	Spermatogonia	Dissociation and filtration of testicular cell (PKH26 labelling)	Yes (1.5 yr)	Yes (2.5 yr)	No	Microinjection into peritoneal cavity of hatchling	Male, 100 (20/20); female, 33.3 (4/12)	Sperm, 66.6 ± 7.6; eggs, 63.2 ± 16.8	Allogenic (yellowtail)	Morita et al. (2012)
Hatchling	Spermatogonia	Dissociation and filtration of testicular cell (PKH26 labelling)	Yes (1 yr)	No	No	Microinjection into peritoneal cavity of hatchling	Male, 2.1 (2/96)	Sperm, 33.3–50	Xenogenic (D, yellowtail; R, jack mackerel)	Morita et al. (2015)
Hatchling	Spermatogonia	GFP positive SG from tg(p*vasa*-GFP)	Yes (2 yr)	Yes (2–3 yr)	Triploid	Microinjection into peritoneal cavity of hatchling	Male, 34.5 (10/29); female, 10 (5/50)	Sperm, 100; eggs, 100	Xenogenic (D,rainbow trout; R, masu salmon)	Okutsu et al. (2007)
Hatchling	Spermatogonia	Dissociation and filtration of testicular germ cells from cryopreserved whole testes	Yes (2–4 yr)	Yes (3–4 yr)	Triploid	Microinjection into peritoneal cavity of hatchling	Male, 100 (7/7)	Sperm, 100; eggs, 100	Allogenic (rainbow trout)	Lee et al. (2013)
Hatchling	Spermatogonia	Dissociation and filtration of testicular cell (PKH26 labelling)	Yes (1–2 yr)	Yes (2 yr)	Triploid	Microinjection into peritoneal cavity of hatchling	Male, 10 (4/40); female, 12.1 (4/33)	Sperm, 100; eggs, 100	Xenogenic (D, Atlantic salmon; R, rainbow trout)	Hattori et al. (2019)
Hatchling	Spermatogonia	Dissociation and filtration of testicular cell (PKH26 labelling)	Yes	Yes	Triploid	Microinjection into peritoneal cavity of hatchling	Male, 36.8 (14/38); female, 28.9 (24/83)	Sperm, 100; eggs, 100	Allogenic (Nibe croaker)	Yoshikawa et al. (2017)
Hatchling	Spermatogonia	Dissociation and filtration of testicular cell (PKH26 labelling)	Yes (11 mo)	Yes (2 yr)	Triploid	Microinjection into peritoneal cavity of hatchling	Male, 38.3 (18/47); female, 31.3 (5/16)	Sperm, 100; eggs, 100	Xenogenic (D, tiger puffer; R, grass puffer)	Hamasaki et al. (2017)
Hatchling	Spermatogonia	Dissociation and filtration of testicular cell from cryopreserved whole testes (PKH26 labelling)	Yes (10 mo)	Yes (2 yr)	Triploid	Microinjection into peritoneal cavity of hatchling	Male, 64.2 (34/53); female, 56.4 (22/39)	Sperm, 100; eggs, 0–100	Xenogenic (D, tiger puffer; R, grass puffer)	Yoshikawa et al. (2018a)
Hatchling	Oogonia	Dissociation and filtration of ovarian germ cells from cryopreserved whole ovaries	Yes (2.5 yr)	Yes (2.5 yr)	Triploid	Microinjection into peritoneal cavity of hatchling	Male, 28 (7/25); female, 20 (5/25)	Sperm, 100; eggs, 100	Allogenic (rainbow trout)	Lee et al. (2016)
Hatchling	Spermatogonia	Dissociation and filtration of testicular cell (PKH26 labelling)	Yes (10 mo)	Yes (2 yr)	*dnd1* KD	Microinjection into peritoneal cavity of hatchling	Male, 91.7 (11/12); female, 26.7 (4/15)	Sperm, 100; eggs, 100	Xenogenic (D, tiger puffer; R, grass puffer)	Yoshikawa et al. (2020)
Hatchling	Spermatogonia	Dissociation and filtration of testicular cell from cryopreserved whole testes (PKH26 labelling)	Yes	Yes	*dnd1* KD	Microinjection into peritoneal cavity of hatchling	Male, 77.8 (14/18); female, 81 (17/21)	Sperm, 66.7–100; eggs, 70.6–100	Allogenic (Chinese rosy bitterling)	Octavera and Yoshizaki (2020)
Hatchling	Spermatogonia	Percoll gradient centrifugation	Yes	Yes	*dnd1* KD	Microinjection into peritoneal cavity of hatchling	Mixed, 43.7 (31/71)	Sperm, 100; eggs, 100	Xenogenic (D, mirror carp; R, goldfish)	Franěk et al. (2021)
Hatchling	Oogonia	in vitro cultured OG for 3 wk. or 6 wk	Yes	No	*dnd1* KD	Microinjection into peritoneal cavity of hatchling	Male, 20 (10/68) for 3 wk. cultured OG; 16 (7/60) for 6 wk.	Sperm, 100	Allogenic (zebrafish)	Wong et al. (2013)
Hatchling	Oogonia	Percoll gradient centrifugation using *tg(vasa:DsRed2-vasa);tg(bactin:EGFP)* double transgenic	Yes (6 mo)	No	Hybrid	Injection into abdominal cavity under the swim bladder	Male, 18 (12/67)	Sperm, 100	Xenogenic (D, zebrafish; R, male pearl danio x female zebrafish hybrid)	Wong et al. (2011)
Hatchling	Spermatogonia	Dissociation and filtration of testicular cell (PKH26 labelling)	Yes (6 mo)	No	Hybrid	Microinjection into peritoneal cavity of hatchling	Male, 100 (43/43)	Sperm, 100	Xenogenic (D, blue drum; R, male white croaker x female blue drum hybrid)	Yoshikawa et al. (2018b)
Adult	Spermatogonia	Percoll gradient centrifugation, differential plating (PKH26 labelling)	Yes (9 wk)	No	Busulfan and heat	Injection through urogenital papilla	Male, 89 (34/38)	Sperm, 6.3 (2/32)	Allogenic Nile tilapia (two strains)	Lacerda et al. (2010)
Adult	Spermatogonia, oogonia	Percoll gradient centrifugation (PKH26 labelling)	Yes (7 mo)	Yes (7 mo)	Busulfan and heat	Injection through genital papilla	Male, 17 (3/17); female 5 (1/20)	Sperm, 12.6–39.7; eggs, 52.2	Xenogenic (D, pejerry; R, Patagonian pejerrey)	Majhi et al. (2014)
Adult	Spermatogonia	Dissociation and filtration of testicular cell (PKH26 labelling)	Yes (7–9 wk)	No	Hybrid	Injection through genital papilla	Male, 10 (4/39)	Sperm, 100	Xenogenic (D, blue drum; R, male white croaker x female blue drum hybrid)	Xu et al. (2019)

a Time: production time for donor derived gametes.

b Frequency of germline chimera formation = number of germline chimera/number of survived adult recipients X 100.

c Germline transmission rate (%) = number of donor-derived hatchlings/number of hatchlings X 100.

The main limitation to the use of PGCs for surrogate technology in several teleost fish is that each embryo has on average only 13–43 PGCs (based on studies including zebrafish (*Danio rerio*), pearl danio (*Danio albolineatus*), loach (*Misgurnus anguillicaudatus*), goldfish (*Carassius auratus*), medaka (*Oryzias latipes*) and ice goby (*Leucopsarion petersii*); Saito et al., 2006). Although a single transplanted PGC can generate germline chimera across species, genus and families (Saito et al., 2008), the low number of PGCs impedes large-scale surrogate production and genetic manipulation. In this context, in vitro culture and growth of PGCs would be necessary to enable the downstream applications. In zebrafish, isolated PGCs from a transgenic line proliferated for over 4 months after optimisation of culture conditions (Fan et al., 2008). However, in most teleost species it is still difficult to culture PGCs reliably, and this is an important area for future research. Another possibility is to generate PGCs in vitro from an embryonic stem cell (ESC) line (Robles et al., 2017). In mammals, where PGCs are specified by inductive signals, PGCs can be stably differentiated from ESCs in vitro by using these signals (Goszczynski et al., 2019). In teleosts, Bivalvia and most Crustracea PGCs are specified by maternal germplasm located in a specific position in the cytoplasm of the zygote. However, despite this inheritance of PGC specification*,* it has been shown that zebrafish PGCs can be differentiated in vitro from embryonic cells by adding growth factors such as retinoic acid, epidermal growth factor, and BMP4. These differentiated PGCs were able to settle in the genital ridge of recipients after transplantation (Riesco et al., 2014). Further developments in in vitro PGC culture systems, improving survival and mitotic activity, are required before these cells can be consistently used in the context of surrogate broodstock technologies.

#### 2.1.2 Gonial cells

Gonial cells (spermatogonia / oogonia) can also be transplanted and settle in the gonadal ridges or gonads of sterilised surrogate species. Despite their gonads are in sexually differentiated state, they show sexual bipotency, and generate sperm or eggs depending on the phenotypic sex of the recipient surrogate animal (Hamasaki et al., 2017; Morita et al., 2012; Okutsu et al., 2007; Wong et al., 2011). The main advantage of gonial cells over PGCs is their abundance; hundreds or even thousands of gonial cells can be obtained from sexually differentiated fish. This abundance also presents an advantage for cryopreservation; whole testes or ovaries can be frozen, and thawed gonial cells produce functional gametes in recipient fish (Lee et al., 2013, Lee et al., 2016; discussed in more detail below). Although PGCs can also be cryopreserved, they require a lengthy procedure and the low number of cells is a limitation (Robles et al., 2017). Although labelling of gonial cells would be useful for isolation and monitoring of the transplanted germ cells, gonial cells can be isolated without labelling procedure from testes or ovaries using their physiochemical and biochemical properties such as size, density and specific receptors (Xie et al., 2020). The simplest way to enrich for gonial cells is to enzymatically dissociate gonads and filtrate the suspension using a filter with a pore size bigger than somatic cells but smaller than gonial cells. This approach has been successfully used in various species for germ cell transplantation (Table 2). The second most commonly used gonial cell enrichment method is density gradient centrifugation (Majhi et al., 2014; Wong et al., 2011). Both of these methods can be combined with differential plating for further elimination of somatic cells based on their adhesive properties to the culture plate (Lacerda et al., 2010; Shikina et al., 2008). Alternative methods to enrich gonial cells include cell sorting based on their light-scattering characteristics (Ichida et al., 2017; Kise et al., 2012), or the use of gonial cell specific antibodies with flow cytometry, FACS or magnetic-activated cell sorting (MACS) (Hayashi et al., 2019; Ichida et al., 2020, Ichida et al., 2019, Ichida et al., 2019). However, it should be noted that these processes may damage gonial cells or alter their physiological features (Xie et al., 2020).

In addition to the putative benefits described above, in vitro culture systems of gonial cells are substantially more advanced than for PGCs, with successful examples of culture of transplantable oogonial (Wong et al., 2013) and spermatogonial cells (Iwasaki-Takahashi et al., 2020; Kawasaki et al., 2016; Shikina and Yoshizaki, 2010) in zebrafish and rainbow trout. Spermatogonial cells are favoured due to their higher abundance, and they can result in either eggs or sperm according to the phenotypic sex of the surrogate recipients. Gonial cell culture requires appropriate sera, feeder cells and growth factors to suppress growth of gonadal somatic cells, promote proliferation of GSCs, and maintain stemness and transplantability of GSCs (Xie et al., 2020). Further studies in other target aquaculture species are needed to determine optimal culture conditions. However, it is clear that the use of gonial cells has advantages over PGCs as donor germ cells; 1) their in vitro culture is simpler and better optimised than for PGCs and 2) they can be isolated in sufficient number to enable the application of surrogate broodstock, even in the absence of a culturing step.

### 2.2 Transplantation into different life stages of sterile surrogate recipients

Isolated germ cells can be transplanted into sterilised recipient animals at different life stages; 1) blastula, 2) hatchlings and 3) adults (Fig. 1). During the blastula stage, germ cells can be transplanted by inserting a graft of donor blastoderm containing PGCs between the blastodermal cells of the recipient (Yamaha et al., 2003), or injecting donor PGCs into marginal region of the blastodisc (Saito et al., 2008) (Table 2, Fig. 1). However, this method requires using PGCs isolated at the early somite stage to achieve migration into the host gonadal ridges (Saito et al., 2008), making this approach impractical for large scale surrogate production due to the limited number of donor PGCs at early stages, and the difficulty in culturing them. Germ cell transplantation is typically conducted at the hatchling stage of the surrogate host. Transplantation at this stage is more conducive to large-scale gamete production from surrogates, since the more numerous gonial cells can be used for transplantation, and they are more amenable to culture (Table 2, Fig. 1). In addition, newly hatched embryos have a relatively immature immune system and therefore this approach is less likely to result in immune rejection of the transplanted germ cells than in an adult recipients (Okutsu et al., 2006). However, injection can be challenging in species with insufficient space in the peritoneal cavity, and blastula stage transplantation might be preferred in these cases (Saito et al., 2008). Finally, gonial cell transplantation at the adult stage of sterile recipient fish through urogenital papilla injection enables production of donor-derived germ cells in a shorter time (Lacerda et al., 2013), but with lower germline transmission rate compared to transplantation at the blastula or hatchling stage (Table 2). Adult broodstock surrogates can be useful for species in which PGC depletion induces masculinization such as zebrafish (Slanchev et al., 2005), medaka (Kurokawa et al., 2007), three-spined stickleback (*Gasterosteus aculeatus*) (Lewis et al., 2008), and Nile tilapia (*Oreochromis niloticus*) (Li et al., 2014), since it would allow recipients of both sexes to be generated for production of both sperm and oocytes.

To enhance surrogate production of donor-derived gametes, independently of the life stage of the surrogate, endogenous germ cells of recipient fish need to be suppressed or ablated as they will outcompete the gametogenesis of donor-derived gametes. Sterilisation strategies include knockdown of essential genes for germ cell development such as *dead end 1* (*dnd1*), triploidy, and inter-species hybrids (Fig. 1). Additionally, adult fish sterility can also be obtained using cytostatic drugs combined with heat exposure.

Triploidisation induces infertility in most teleost species which can be obtained by physical or chemical shock during meiosis II (Piferrer et al., 2009) and has been widely used for surrogate production in several species (Table 2). However, triploid individuals can be fertile in certain species, especially those in which triploids can occur naturally (i.e. Prussian carp and *Cobitis* spp.; Piferrer et al., 2009), while in others sterility can induce monosex populations (e.g. bitterling; Octavera and Yoshizaki, 2019) or induce the production of a large proportion of aneuploid eggs (e.g. grass puffer; Hamasaki et al., 2017). Additionally, triploidisation or similar strategies are only practical in diploid species, as for example individuals with 1.5 fold increment of chromosome sets of the tetraploid species Siberian sturgeon (*Acipenser baerii*) are fertile (Havelka et al., 2014). Interspecies hybrids are also typically sterile and have been used as germ cell recipients in various species (Kawamura et al., 2020; Piva et al., 2018; Wong et al., 2011; Yamaha et al., 2003; Yoshikawa et al., 2018). However, this method will be limited to certain species which have available sterile hybrids such as blue drum (Yoshikawa et al., 2018) and mackerel (Kawamura et al., 2020). Therefore, both triploidisation and hybridisation have limited utility for achieving sterile surrogate hosts.

A highly promising and widely used method to produce sterile recipients is the knockdown or knockout of *dnd1* or other genes playing essential roles in PGC survival and migration (Weidinger et al., 2003). Knockdown or knockout of *dnd1* can be achieved by microinjection of *dnd1* antisense morpholino oligomers (MO) or genome editing tools such as CRISPR/Cas9 into zygotes (discussed in more detail below). This approach has successfully produced sterile embryos in various species including zebrafish (Saito et al., 2008), masu salmon, rainbow trout (Yoshizaki et al., 2016), Atlantic salmon (Wargelius et al., 2016), sterlet (*A. ruthenus*; Linhartová et al., 2015), grass puffer (Yoshikawa et al., 2020) and Chinese rosy bitterling (Octavera and Yoshizaki, 2019). Furthermore, germ cells in *dnd1* KO salmon could be rescued by co-injecting the salmon *dnd1* mRNA together with Cas9/gRNA targeting *dnd1* (Güralp et al., 2020). This strategy would allow fertile surrogate broodstocks that produce sterile offspring.

While hatchling transplantation of germ cells is typically most practical, there are species where it is implausible. For example, species where PGC depletion induces masculinization, gynogenetic polyploids such as the cyprinid *Carassius gibelio* (Liu et al., 2015), or sequential hermaphroditic species (Table 1). In these cases, adult surrogates may be necessary to achieve both male and female gamete production, even though germ cell transmission is low compared to other stages. The most commonly used sterilisation method for sexually competent recipient fish is treatment with the cytostatic drug busulfan, followed by exposure to heat (Nile tilapia, Lacerda et al., 2010; Patagonian pejerrey, Majhi et al., 2014). This strategy is only feasible in species where immunological rejection is not triggered in adult recipient animals, with potential preference for transplantation of spermatogonia over oogonia due to the immune privileged lumen in testes (Batlouni et al., 2009; de Siqueira-Silva et al., 2018).

## 3 Surrogate technology to improve genome editing research in aquaculture

The use of surrogate broodstock technology has major potential to enhance the scope and efficiency of genome editing research in aquaculture species. The advent of CRISPR/Cas genome editing systems has accelerated gene and genome function research through the generation of animals and cell lines carrying precise targeted edits. In aquaculture species, this has typically been performed by pronuclear microinjection of genome editing molecules in early-stage embryos, which has been successful in achieving gene knockout in the founder (F0) animals (reviewed in Gratacap et al., 2019). However, this process inevitably generates mosaic founders, which are not ideal for assessing the phenotypic consequences of the edits (Mehravar et al., 2019). There are two types of mosaicism in founder animals: 1) unevenly distributed edited and wild-type cells across tissues and organs, and 2) edited cell populations carrying multiple different edited alleles. Both of these represent major issues for assessing the consequence of targeted editing, and the long generation interval of most species means that crossing of founders to achieve homozygous F1 animals is practically challenging, and doesn't fully address the issue of mosaicism for different edited alleles. The use of genome editing in germ cells, in combination with surrogate broodstock technology, has potential to overcome these issues.

Direct genome editing of GSCs has not yet been reported in aquaculture species, but is effective in chicken and frogs where edited PGCs were transplanted into recipients and produced homozygous F1 and F2 progeny, respectively (Blitz et al., 2016; Oishi et al., 2016). Individual progeny should in theory carry a target edit in all cells, preventing the occurrence of mosaicism in founders. Furthermore, screening for specific edits and intercrossing of surrogates carrying donor-derived germ cells with identical edits can ensure that progeny are homozygous for the target edits. The main caveat of this approach is the need to wait until the surrogates are sexually mature, but in turn it allows the production of fully edited homozygous animals. These animals could then be taken forward for phenotypic characterisation, and potentially application in commercial breeding programmes. This approach is likely to be particularly beneficial for later-stage genome editing research, for example where mosaic editing has highlighted the importance of a particular gene knockout, and the goal is to provide a detailed characterisation of a specific edited allele.

### 3.1 Performing genome-scale CRISPR/Cas9 knockout (GeCKO) screens in vivo

**Fig. 2:**
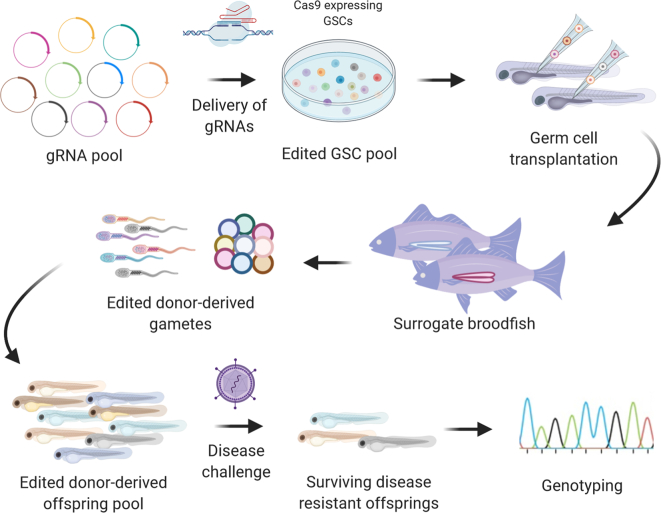
Schematic overview of early-life in vivo genome-wide CRISPR screening for disease resistance by targeting GSCs and subsequent transplantation using surrogate technology in fish.

Genome-wide CRISPR Knock-Out (GeCKO) experiments are feasible in fish cell lines that are amenable to lentiviral transduction, such as Chinook salmon CHSE-214 (Gratacap et al., 2019), and allow the perturbation of thousands of genes or other genomic features simultaneously (Shalem et al., 2014). However, while useful particularly for traits such as resistance to pathogens, the use of cell lines offers limited information on many traits of interest, which may not correlate with the results at the whole organism level. Unlike mammals, many aquaculture species show high fecundity of both sexes, which in theory makes possible the development of in vivo GeCKO screens, especially if combined with surrogate broodstock technologies. For example, GSCs in culture could be engineered to express the CRISPR/Cas system, with genome-wide gRNA pools delivered by viral vectors or transposons to the engineered Cas-expressing GSCs. Subsequently, the edited GSC pool can be transplanted into surrogate broodstock (Fig. 2). The surrogates will then produce pools of gametes carrying edits for different targets (e.g. each individual gamete would carry an edit for a single gene, but in theory the pool would contain gametes with edits for every genomic locus). If the expression of the Cas effector/gRNA continues throughout fertilisation the other gamete's DNA would also be edited, likely resulting in the knockout of the two copies of the target gene. For example, chicken PCGs engineered to express Cas9 using the Tol2 transposon in vivo could generate biallelic mutant F1 when crossed with wild-type showing stable expression of Cas9 (Challagulla et al., 2020). Thus, when these GeCKO gamete-producing surrogate fish are crossed with wild type fish, pools of offspring carrying biallelic mutations for each gene could theoretically be produced. In addition, intercrossing between the animals bearing pools of edited gametes would typically result in offspring carrying edits at two loci, which in a large pool of offspring would include many combinations of pair-wise knockouts throughout the genome. This raises the interesting possibility of genome-wide in vivo epistatic screenings (i.e. carrying different combinations of biallelic mutations), which would allow for detection of putative epistatic effects on phenotypes of interest.

There are several critical steps for this ‘thought experiment’ to become reality, which include engineering Cas-expressing GSCs, and the efficient delivery of pooled gRNA libraries to GSCs. Successful GSC transgenesis has been reported using lentiviral, adeno-associated viral transduction, piggyBac- or Tol2 transposon-mediated gene transfer in rat, mouse, pigs and chicken, generating transgenic offspring (Park and Han, 2012; Ryu et al., 2007; Watanabe et al., 2018; Zeng et al., 2013). These results suggest that viral vectors or piggyBac- or Tol2 transposon-mediated transgenesis of piscine GSCs is feasible, both for delivery of gRNA libraries and generation of engineered Cas9-expressing GSCs. Notably, integration of CRISPR/Cas system is reversable with transposon-mediated genome engineering such as piggyBac or Tol2 for the removal of the transgenes which is important for commercial application (Challagulla et al., 2020). Finally, in addition to standard Cas enzymes, catalytically inactive Cas9 (dCas9)-based CRISPR interference (CRISPRi) or CRISPR activation (CRISPRa) screening platforms could enable mapping of genetic interaction or epistatic screening in vivo (Du et al., 2017).

## 4 Applications for improved breeding and production of aquaculture stocks

**Fig. 3:**
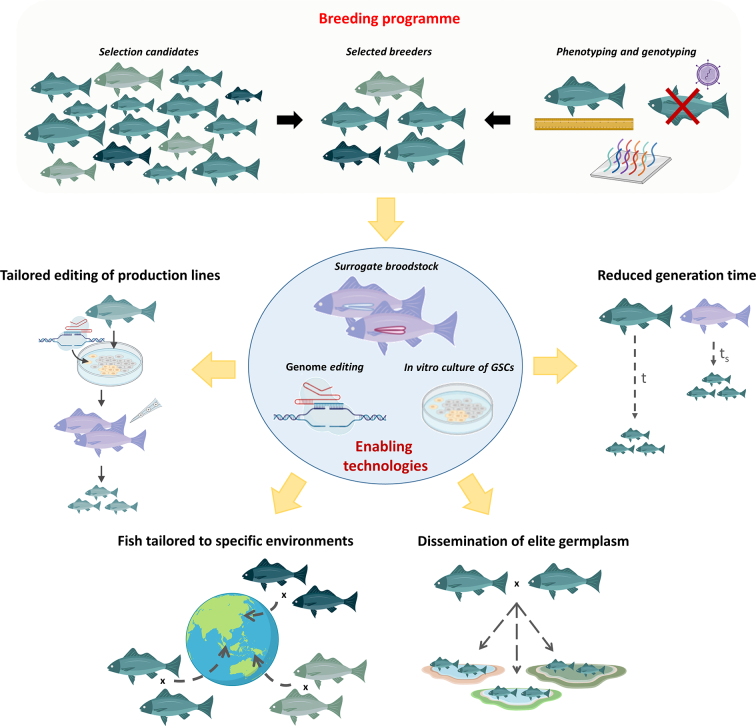
Overview of four potential applications of surrogate broodstock and GSC editing technologies for improved breeding and production in aquaculture.

The aforementioned examples of integrating surrogate broodstock and genome editing are primarily focussed on an experimental research tool. However, surrogate broodstock technologies also have possible applications in aquaculture breeding programmes and production settings to expedite genetic improvement and dissemination of elite germplasm. The combination of these surrogate technologies with genomic selection, and potentially also genome editing, could drastically increase genetic gain. Below we describe several different strategies to potentially exploit surrogate broodstock technologies in aquaculture breeding programmes (Fig. 3).

### 4.1 Overcoming barriers to aquaculture and conservation of high value species

There are several aquaculture species for which the full lifecycle cannot be completed in captivity, and genetic material is therefore sourced from the wild. This can be for reasons such as lack of control of reproduction in captivity, or lack of knowledge of suitable conditions for rearing early-stage juveniles. By isolating GSCs from those species, in vitro expansion and cryopreservation, the genetic materials can be reliably sourced. Then, transplantation into surrogate broodstocks which have shorter generation time with amenable reproductive cycle in captivity could reliably produce gametes of species which are challenging to rear in captivity. For example, species with unreliable spawning, sterlet sturgeon females, not only take up to 9 years to reach sexual maturity, but also tend not to spawn every year. Similar approaches could be applied to avoid exposure to the marine phase in broodstock of anadromous species, such as Atlantic salmon, which requires a potentially stressful transition from freshwater to seawater and results in a prolonged exposure to a more hazardous environment. For example, Atlantic salmon PGCs could be transferred to the riverine ecotype of brown trout, which does not have a marine phase. In addition, surrogate broodstock technology has great potential as a tool to preserve endangered species. For example, xenogenic transplantation of germ cells from endangered species has been reported in several species such as Chinese sturgeon (*Acipenser sinensis*) into Dabry's sturgeon (Ye et al., 2017), Caspian trout (*Salmo caspius*) into rainbow trout (Poursaeid et al., 2020), and *Brycon orbignyanus* into *Astyanax altiparanae* (de Siqueira-Silva et al., 2019). However, as yet, production of donor-derived offspring from the surrogates has not been reported.

### 4.2 Cross-species surrogate broodstock technology to reduce generation interval

Many aquaculture species have long generation times. For example, Atlantic salmon usually take 4 years to sexually mature, Pacific oysters reach sexual maturity between 2 and 3 years post hatching, and Bluefin tuna can take up to 5 years (Table 1). These long generation intervals limit the genetic gain that can be achieved through selective breeding. Consequently, the application of surrogates via transplantation of gametes from a donor species with a long generation interval to recipients of species with a shorter one can significantly accelerate genetic gain and stock improvement. As shown in the breeders' equation:

$$R_{t}=\frac{i\;r\;\sigma_{A}}{t}$$

where *R*_*t*_ is genetic gain over time, *i* is selection intensity, *r* is selection accuracy, *σ*_*A*_ is additive genetic variance, and *t* is generation time. Putting aside certain logistical and practical constraints, reductions in generation time are directly translated into increases of the same magnitude in the rate of genetic gain – i.e. 50% shorter generation time leads to 50% increased rate of genetic gain. When compared to the relatively small incremental genetic gain that is derived from increasing selection accuracy via improvements in genomic selection methodology (for example), it is clear that investigating the potential of surrogate broodstock technology to achieve these gains is a worthy exercise, particularly for high value species with long generation intervals.

There are several practical aspects to be considered in relation to this concept, beginning with which of the aforementioned germ cell isolation, sterilisation, and transplantation methods and timings would fit within the context of a breeding programme. If we assume that there are available sterile xenogenic recipient species with a shorter generation time than the donor species, and that sufficient GSCs can be obtained from the donor broodstock, there are three potentially relevant methods to produce donor-derived offspring using different stages of donor and recipient animals: 1) PGCs from donor embryos to xenogenic recipient embryos, 2) gonial cells from sexually differentiated donor to recipient embryos or to 3) adult recipients.

Considering the relative simplicity of isolating gonial cells, the abundance and the advanced in vitro culture system compared to PGCs (as described in section 2), gonial cells are likely to be preferred as donor GSCs. Although it takes a longer time for donor animals to have sexually differentiated gonads compared to PGC isolation, this can allow time for direct genotyping and phenotyping (for certain traits) of the donor animals and their full siblings, as discussed below. Regarding the developmental stage of recipients, adult stage recipients can greatly reduce the generation time; adult male surrogates would be particularly desirable since the testes are considered an immune privileged tissue (Batlouni et al., 2009), however, there is a high probability of immune rejection in the ovary of adult females, which can be a limiting factor. Taking these factors into consideration, the optimal approach may be transplantation of gonial cells from sexually differentiated donor juveniles to sterilised recipient embryos.

**Fig. 4:**
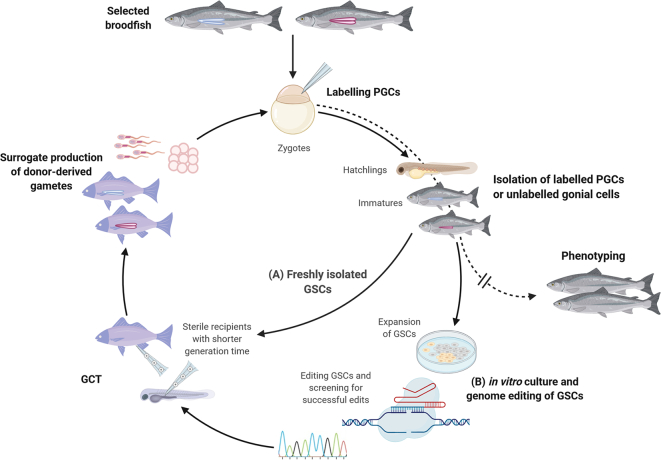
Accelerating genetic gains with reduced generation interval by using in vitro expansion and genome editing of GSCs and surrogate broodstocks. To isolate GSCs, zygotes produced from selected broodfish can be microinjected with PGC labelling constructs to isolate PGCs at embryonic or hatchling stages while unlabelled gonial cells can be isolated from sexually differentiated fish (immature or mature). (A) Freshly isolated GSCs can be transplanted into the recipients with shorter generation time to produce donor-derived progeny in a shorter time. (B) The isolated GSCs can be also expanded in vitro and edited to improve specific traits using genome editing tools such as CRISPR/Cas system. GSCs with desired edits can be screened and transplanted into sterile recipients to produce the progeny with improved traits. Since it loses genetic accuracy every generation, phenotyping and evaluating of breeding values will be required at regular intervals by letting some of the progeny to mature (dotted line).

The process would begin with generating donor animals by crossing selected broodstock animals as would be routine in a breeding programme. Then, when their offspring reach the stage suitable for tissue sampling (i.e. a small amount of tissue can be excised for genotyping without negative impact on the fish welfare), they would be genotyped and their genomic breeding values would be estimated (see subsequent paragraph and Fig. 4). Gonial cells from the top ranked selection candidates (i.e. those with the top genomic breeding values for target traits) could then be isolated. At this stage, the donor cells could be expanded in vitro, and potentially genome edited and screened if desirable (see section below). In parallel, sterile xenogenic recipients with shorter generation interval would be generated, and the donor germ cells of the donors transplanted into their peritoneal cavity during hatchling stages. The progeny of the surrogates would then be used as potential donors for the next round of selection (Fig. 4).

The use of genomic selection in this process is key, since it enables accurate prediction of the genetic merit of animals early in their lifecycle based on their DNA, without the need for trait measurements on the individuals themselves. Early life selection is challenging for many traits (i.e. sea lice resistance in Atlantic salmon, which is only measurable in the sea water stage of their life cycle). For these phenotypes, the training population for the genomic prediction model would likely be based on the phenotypes of previous generations. However, that would likely result in a reduction of the selection accuracy (r), and therefore of genetic gain, compared to the standard ‘sib-testing’ approach (Zenger et al., 2019). The trade-off between accelerating generation time and reducing selection accuracy would need to be assessed in those species with traits of interest not measurable early in the lifecycle prior to any application of surrogate technologies.

An additional practical concern for this proposed scheme would be the requirement to maintain breeding lines of the surrogate species, in addition to the target food production species (e.g. surrogate trout lines as for Atlantic salmon breeding). This would be a significant additional cost and presents major logistical and biosecurity considerations. For this surrogate species, the shorter the generation interval, the more rapid the increase in genetic gain could potentially be. Therefore, further reduction in the generation interval could be achieved via environmental manipulations (photoperiod, temperature), or genetic selection. It is important to note that time to maturity and the possibilities for environmental manipulation will vary according to sex in several species, such as the precocious maturity possible within a year in Atlantic salmon males. In addition, while genetic variation in time to sexual maturity exists and therefore generation time could be reduced via selective breeding, this is usually not desired in aquaculture; sexually mature animals tend to grow slower and can have several other undesirable characteristics (Iversen et al., 2016). In fact, the use of surrogate broodstock would allow selection for late maturity in the donor species, decreasing associated problems and further increasing aquaculture productivity and fish health. On the contrary, the surrogate species could be selected for early sexual maturation to amplify the benefits of the technology, further increasing the rate of genetic gain. This would require the running of a second concurrent breeding programme, and while it would be simple (e.g. maintaining genetic diversity, reducing generation time) it would be a significant additional cost.

### 4.3 Dissemination of elite germplasm to farmers

Surrogate broodstock technologies can also increase the genetic merit of production animals through widespread dissemination of elite germplasm, with a result in improved average production performance. Typically, producers would receive germplasm that is some distance removed from the elite animals with the highest genetic merit, due to the need to produce large numbers of animals for production. This issue is well known in breeding programmes for terrestrial farmed species such as chickens and pigs, where breeding nuclei lie at the top of a substantial pyramid separated by several layers of multiplication. However, it also occurs in some aquaculture breeding programmes, especially where multiplier layers, including local breeding hatcheries, are used to provide eggs to farmers. With the concept of surrogate broodstock, in principle, all production animals could be generated from a single cross, or a few crosses, of the best males and best females in the population. The germ cells of these animals could be transplanted to sufficient numbers of surrogates (of the same or other species) to generate all production animals (albeit the need for genetic diversity in farmed populations would need to be considered with regard to risks of disease outbreaks, for example). While the benefits are even larger with more multiplication layers, reducing the genetic lag between nucleus and production animals (Gottardo et al., 2019), in any case the genetic merit of the production animals will be substantially higher than in a standard breeding scheme. However, while shortening generation time produces cumulative genetic gains over generations, reducing genetic lag only improves the genetic merit of the current production animals and is not propagated to future generations. In practical terms, it can be considered that the genetic merit of each generation is increased by a constant value. Nonetheless, being able to generate the best possible production animals is likely to have a major impact in the performance of aquaculture species.

### 4.4 Improved tailoring of production fish genetics to specific environments

The previous concept can also be extended to generate the best production animals for specific production situations or environments. Considering breeding programmes usually utilise breeding indexes composed of different traits with varying economic value, surrogate broodstock technologies would allow maximising a trait or sub-index of interest in the production animals according to the current needs of the farms without compromising the long-term breeding plan. This is analogous to placing intense selection pressure on specific traits or sub-indices in multiplier animals, which is currently practiced in some large-scale salmon breeding and production programmes. Similarly, surrogate broodstock could facilitate the adaptation of a single stock to different farms and environments; aquaculture breeding programmes are progressively becoming centralised, meaning animals are disseminated from a single hatchery to various farms distributed around the globe. In this context, the ability to adapt to the needs of each production environment, and potentially have a better control over GxE interactions, can lead to significant improvements in the health and performance of the production animals.

Pending acceptable regulatory and public perception environments, selection of the best elite broodstock for dissemination could also be coupled with genome editing to further adapt production animals to overcome production barriers related to particular environmental conditions. Surrogate broodstock facilitates the production of progeny carrying desired edits as described above, and enables production of different combinations of edits in different groups of full siblings. This approach could be especially effective to tackle temporal variation in environmental conditions. For instance, seasonal variations or disease outbreaks, when specific edits that might typically be detrimental for other production traits will be desirable. Therefore, such edits would not be desirable in the broodstock of a long term breeding programme, but tailored editing of germ cells before mass production via surrogates means the impact of the edits are temporary. The combination of genome editing and surrogate broodstock can be extremely powerful, but basic research is necessary to find the right genetic targets and, of course, regulatory hurdles are still in place in most aquaculture producing countries. This is likely to be particularly relevant in aquaculture systems where interbreeding with wild conspecifics is likely, for example via escapees. Surrgoate broodstock technologies may be a solution to this issue via mass production of sterilised animals as discussed below. Furthermore, if the editing is performed in the germ cells prior to transfer to surrogates, then this may facilitate dissemination of both edited and non-edited production animals according to the variations in regulatory landscape.

Importantly, genome editing can also be used in combination with surrogate broodstock to produce sterile production animals that, even if they escape, won't reproduce and alter the genetic makeup of wild populations. This would contribute to diminishing the environmental impact of aquaculture and safeguard natural stocks, and may also contribute towards the global acceptance of genome editing in aquaculture and more permissive legislations. It is likely that any use of genome editing in commercial aquaculture stocks to improve production traits (e.g. disease resistance) will need to be disseminated for production in sterile animals. Including the trait of sterilisation in a breeding programme is challenging for obvious reasons, but with the surrogate broodstock mediated editing and dissemination, sterilisation via (e.g.) *dnd1* knockout could easily be incorporated alongside other desired edits for production.

## 5 Conclusions

Genomics and biotechnology advances have a major role to play in supporting genetic improvement of aquaculture species, and in turn global food security. Surrogate broodstock research has been used successfully in several finfish species to enable production of donor-derived gametes in sterilised surrogate hosts of the same or a related species. Advances in germ cell culture is required to improve the efficiency of this process, and the use of gonial cells has advantages over PGCs due to their abundance and relative ease of culture. Methods of sterilisation of recipients have advanced rapidly, and CRISPR knockout of essential fertility genes such as *dnd1* is highly effective. As surrogate broodstock technology advances, it provides new opportunities for genome editing research and application via CRISPR editing in cultured germ cells. This is likely to reduce mosaicism, and offer potential for genome-wide CRISPR screens in edited progeny of surrogate broodstock as a research tool. Furthermore, surrogate broodstock technology offers potential for aquaculture production, including expediting genetic gain via use of surrogate species with shorter generation interval in breeding programmes. The technology can also support widespread dissemination of elite germplasm, potentially including sterile animals edited for desirable alleles for production traits, for aquaculture production. This provides a route to application of genome editing in aquaculture without incorporating edits directly into the breeding nucleus animals, supporting temporal and tailored use of CRISPR technology. Finally, surrogate broodstock technology can support conservation of valuable aquatic genetic resources, and enable reproduction of high-value aquaculture species which are otherwise difficult to rear in captivity. While significant research is still required, the synergy of surrogate broodstock, genomic selection, and genome editing has potential to transform aquaculture research and production in the coming decades.

## Declaration of Competing Interest

I confirm that none of the authors of the manuscript have any conflicts of interest.
